# Residential Green and Blue Spaces and Type 2 Diabetes Mellitus: A Population-Based Health Study in China

**DOI:** 10.3390/toxics9010011

**Published:** 2021-01-16

**Authors:** Ruijia Li, Gongbo Chen, Anqi Jiao, Yuanan Lu, Yuming Guo, Shanshan Li, Chongjian Wang, Hao Xiang

**Affiliations:** 1 Department of Global Health, School of Health Sciences, Wuhan University, Wuhan 430071, China; ruijia@whu.edu.cn (R.L.); anqi_jiao97@163.com (A.J.); 2 Global Health Institute, Wuhan University, Wuhan 430071, China; 3 Guangdong Environmental and Health Risk Assessment Engineering Technology Research Center, Department of Occupational and Environmental Hygiene, School of Public Health, Sun Yat-sen University, Guangzhou 510080, China; chengb36@mail.sysu.edu.cn; 4 Environmental Health Laboratory, Department of Public Health Sciences, University Hawaii at Manoa, Honolulu, HI 96822, USA; yuanan@hawaii.edu; 5 Department of Epidemiology and Biostatistics, School of Public Health, Zhengzhou University, Zhengzhou 450001, China; yuming.guo@monash.edu; 6 Department of Epidemiology and Preventive Medicine, School of Public Health and Preventive Medicine, Monash University, Melbourne, VIC 3010, Australia; Shanshan.Li@monash.edu

**Keywords:** residential environment, green space, water body, type 2 diabetes

## Abstract

Evidence on the health benefits of green space in residential environments is still limited, and few studies have investigated the potential association between blue space and type 2 diabetes mellitus (T2DM) prevalence. This study included 39,019 participants who had completed the baseline survey from the Henan Rural Cohort Study, 2015–2017. The Normalized Difference Vegetation Index (NDVI) and Enhanced Vegetation Index (EVI) were employed to characterize the residential green space, and the distance from the participant’s residential address to the nearest water body was considered to represent the residential blue space. Mixed effect models were applied to evaluate the associations of the residential environment with T2DM and fasting blood glucose (FBG) levels. An interquartile range (IQR) increase in NDVI and EVI was significantly associated with a 13.4% (odds ratio (OR): 0.866, 95% Confidence interval (CI): 0.830,0.903) and 14.2% (OR: 0.858, 95% CI: 0.817,0.901) decreased risk of T2DM, respectively. The residential green space was associated with lower fasting blood glucose levels in men (%change, −2.060 in men vs. −0.972 in women) and the elderly (%change, −1.696 in elderly vs. −1.268 in young people). Additionally, people who lived more than 5 km from the water body had a 15.7% lower risk of T2DM (OR: 0.843, 95% CI: 0.770,0.923) and 1.829% lower fasting blood glucose levels (95% CI: −2.335%,−1.320%) than those who lived closer to the blue space. Our findings suggest that residential green space was beneficially associated with T2DM and fasting blood glucose levels. However, further research is needed to explore more comprehensively the relationship between residential blue space and public health.

## 1. Introduction

Rapid urbanization has caused a large number of problems, such as air pollution, reduced green vegetation, and depleted water resources [1]. Growing attention is currently being paid to the role of the totality of environmental exposures and their endogenous response as it is imprinted across the lifespan in shaping disease risk and disease development [2]. In this respect, residential green and blue spaces were found to regulate ecosystem services, improve air quality, reduce heat island effects, and diminish noise pollution [3,4]. Meanwhile, they can also promote overall public health by increasing physical activity, improving self-perceived health, and lowering the incidence of respiratory diseases [5,6].

Environmental intervention is considered one of the most effective upper-level health management measures for chronic cardiovascular and cerebrovascular diseases [7,8]. The association between abundant residential green space and the lower risk of type 2 diabetes mellitus (T2DM) has been observed in earlier studies [9,10,11,12,13,14,15,16,17]. For example, Ngom et al. found that the prevalence of T2DM was 9% higher in people whose homes were the furthest from green spaces than in those who lived in their nearest proximity [18]. Clark et al. found that an interquartile range (IQR) increase in greenness was associated with a 10% reduction in the prevalence of T2DM (odds ratio (OR): 0.90, 95% confidence interval (CI): 0.87,0.92) [19]. However, Lee et al. and Afroz-Hossain et al. showed that the long-term beneficial association between green space and T2DM was not consistent [20,21].

A growing number of studies have explored the beneficial effects of residential green space, but relatively little attention has been paid to blue space [22]. Only one cohort study discovered that living within 250 m of a water body was associated with a 12%–17% lower risk of mortality, excluding the effects of external/accidental causes [23]. High-quality epidemiological studies were scarce to explore the association between residential blue space and T2DM prevalence. In addition, due to the lack of high-quality residential exposure data and detailed baseline population information, certain potential confounding factors have not been controlled in previous studies [24,25]. 

Therefore, in the present study, we aimed to evaluate the associations of residential environment (including green and blue spaces) with T2DM and fasting blood glucose (FBG) levels in a Chinese population. Furthermore, we investigated the potential underlying mechanisms in this association, and performed multiple sensitivity analyses to confirm the robustness of our results.

## 2. Materials and Methods

### 2.1. Study Population

All participants were from the Henan Rural Cohort Study (Registration number: ChiCTR-OOC-15006699), established in five rural areas of Henan Province, China, during the period from 2015 to 2017. Its main objective was to examine the burden of multiple chronic diseases in the population, explore potential risk factors, clarify trends in non-communicable diseases, and develop disease risk scoring models suitable for Chinese people. Detailed descriptions of the cohort design and study population were previously published elsewhere [26,27]. 

A total number of 39,259 participants in the Henan Rural Cohort Study were recruited, and each participant signed an informed consent form. After excluding participants who did not accept blood glucose measurement and those who lacked other vital data, 39,019 participants were actually available for analyses. 

### 2.2. Residential Green and Blue Space Assessments

Unlike the early epidemiological method, which only considered the reductionist concept of “one exposure, one result” associations at a time, the new exposure process also included examining the multiple environmental exposures of individuals and populations that dynamically change over time and space. Therefore, the more abundant exposure assessment tools and indicators are used, the more accurate the measurement of the exposome will be [2,28].

In our research, the Normalized Difference Vegetation Index (NDVI) and the Enhanced Vegetation Index (EVI) were employed to characterize the residential green space. These two vegetation indices are derived from satellite images, and obtained by the Moderate Resolution Imaging Spectroradiometer (MODIS) [29]. NDVI is a widely used index describing the relative overall vegetation density and quality, which is calculated based on land surface reflectance of near-infrared (NIR) and visible red (VR) wavelengths. EVI is similar to NDVI, but a blue light band (BLUE) is introduced to correct the atmospheric and soil background [30]. The values of both vegetation indices ranged from −1 to 1, with negative values representing water, zero value denoting bare soil, and higher, positive values indicating dense green vegetation. We calculated the average green exposure of each participant in the 500 m buffer zone around their residential address within three years before inclusion in the cohort. The 500 m buffer zone was selected to represent the direct environment around the home, which is potentially more relevant for people.

The process of calculating the blue space distance consisted of the following two steps. First, we defined the closest distance between the participant’s residence and the water body as the blue space distance. Second, we calculated the distance from residential address to the nearest river by the Nearest tool in Arc GIS 10.2, and Figure S1 (Supplementary Materials) shows the specific process of calculating the blue space distance.

**Figure 1:**
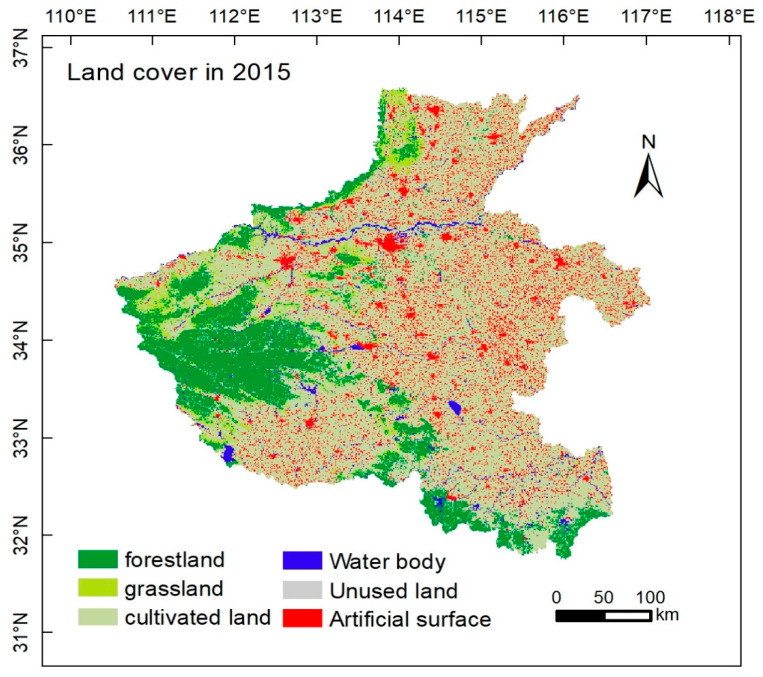
Remote sensing map of land cover in research area.

We divided land use types into six types based on the Land Use and Cover Change (LUCC) classification system: forestland, grassland, cultivated land, water area, artificial surface, and unused land. The land use data came from the Resource and Environmental Science and Data Center of the Chinese Academy of Sciences (http://www.resdc.cn/), with a spatial resolution of 1 km (Figure 1). 

### 2.3. Outcome Assessments

Following the Clinical Chemistry Blood Sample Collection and Processing guidelines [31], we collected blood samples from participants who had fasted for at least eight hours. The samples were immediately sent to the laboratory for serum and plasma separation. A Roche Cobas c501 (Switzerland, Basel) analyzer was used for testing of the fresh serum specimens to determine the fasting blood glucose levels of the participants. 

Following the recommendations of the International Classification of Diabetes Mellitus Diagnostics [32], we diagnosed T2DM in all subjects that met any of the following three criteria: (1) defects in insulin secretion or action of the participants were not caused by type 1 diabetes mellitus and gestational diabetes mellitus; (2) the patient had been diagnosed with T2DM or had been taking antidiabetic drugs directed by doctors; and (3) the fasting blood glucose level exceeded 7.0 mmol/L. 

### 2.4. Covariates

Based on the evidence in the existing literature, we selected some variables as covariates [33]. Demographic covariates included age (years) and sex (male, female). Additionally, health status covariates were assessed, including body mass index (BMI) (kg/m^2^) and family history of diabetes mellitus (no, yes). The following socioeconomic covariates were examined: marital status (married/living together, divorced/widowed/separated/unmarried), education level (no school or primary school, middle school, junior college or higher), and monthly income level (low, medium, or high). We also tested the following health behavior covariates: smoking status (never, former, or current), drinking status (never, former, or current), high-fat diet (average consumptions of meat from livestock and poultry by each participant of more than 75 g per day) (no, yes), fruit and vegetable intake (average intake of fruits and vegetables by each participant of more than 500 g per day) (no, yes), and physical activity (low, medium, or high) [34]. 

### 2.5. Statistical Analyses

After cleaning up the cohort’s baseline data and environmental exposure data, we described age, sex, fasting blood glucose level, residential green space, and other characteristics of all included participants. We explored the relationship between the residential environment and fasting blood glucose levels through scatter plots and performed a natural logarithmic conversion of the fasting glucose levels to improve the normality before the regression analysis. Mixed effect models were implemented to elucidate the effects of residential environment on T2DM and fasting blood glucose levels. Three types of models (Crude model, Model 1, and Model 2) were performed in our analyses. A minimal set of potential confounders was adjusted in Model 1: age, sex, BMI, monthly income, and physical activity. Model 2 was further adjusted for education level, marital status, smoking, drinking, high-fat diet, fruit and vegetable intake, and family history of diabetes. Then, we analyzed the interaction effects through adding confounding factors as cross-product terms. The analysis results were presented as OR value and percentage change (%change), respectively. 

To examine the robustness of our results, we conducted a series of sensitivity analyses by removing subjects with a family history of diabetes, hypertension, or dyslipidemias. Different green exposure time values (e.g., 1-, 2-, and 4-year average values) and buffer radius (e.g., 1000 m, 3000 m) were replaced to test the sensitivity of the results. All statistical analyses were completed using R 3.6.1 (https://rstudio.com).

## 3. Results

The basic characteristics of all participants are presented in Table 1. The mean age of all participants was 55.58 ± 12.18, and the majority of them were female (60.6%). Overall, 3674 (9.4%) subjects were diagnosed with T2DM, the number of self-reported patients was more than those diagnosed by blood glucose measurement (58.1% vs. 41.9%). Compared with participants without T2DM, those with T2DM were relatively older (60.35 years vs. 55.10 years), with a higher BMI (26.18 kg/m^2^ vs. 24.69 kg/m^2^), higher monthly income (31.8% vs. 28.5%), and higher family history of T2DM (9.9% vs. 3.6%). The mean (SD) values of NDVI and EVI within a 500 m buffer radius were 0.48 (0.07) and 0.34 (0.06), respectively. As shown in Table S1, the prevalence of T2DM among people living in relatively low-green areas was 11.0%, and it is 6.6% and 9.2% in high-green areas, respectively. 

In crude and adjusted models, the availability of more residential green spaces was associated with lower T2DM prevalence and fasting blood glucose levels (Table 2). When the residential green space was considered as a categorical variable, the risk of T2DM was 13.5% (95% CI: 12.8%, 14.5%) lower with exposure to the highest quartile (Q4) of NDVI compared with those exposed to the third quartile (Q3) (Model 1). Associations between residential green space and fasting blood glucose levels showed the same similar results with T2DM prevalence. When the residential green space was considered as a continuous variable, in the fully adjusted model (Model 2), the IQR increase in NDVI and EVI was significantly associated with a 13.4% (95% CI: 9.7%,17.0%) and 14.2% (95% CI: 9.1%,18.3%) decrease in the risk of T2DM, respectively. Moreover, 1.384% (95% CI: −1.726%,−1.040%) and 1.273% (95% CI: −1.672%,−0.871%) lower fasting blood glucose levels were also observed with IQR increase in NDVI and EVI, respectively. 

**Figure 2:**
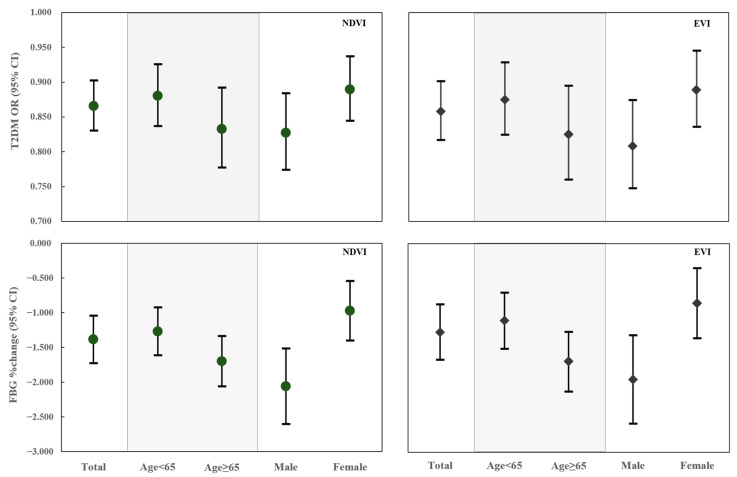
Interaction effects of potential confounders in associations between residential green space and type 2 diabetes mellitus and fasting blood glucose levels. Models were adjusted for age, sex, body mass index, education level, marital status, monthly income, smoking, drinking, high-fat diet, fruit and vegetable intake, physical activity, and family history of diabetes.

Interestingly, the residential green space was associated with lower fasting blood glucose levels in men (%change, −2.060 in men vs. −0.972 in women) and the elderly (%change, −1.696 in elderly vs. −1.268 in young people) (Figure 2, Table 3). Participants with normal weight were more affected by the residential green space, but it was not statistically significant. No obvious moderating effects of different levels of monthly income and physical activity were observed.

Models were adjusted for age, sex, body mass index, education level, marital status, monthly income, smoking, drinking, high-fat diet, fruit and vegetable intake, physical activity, and family history of diabetes. The distance between the residential area and the nearest water body was also related to the risk of T2DM. In the Crude model, the accessibility of the residential blue spaces was associated with lower T2DM risk (OR: 0.884, 95% CI: 0.837,0.934) and fasting blood glucose levels (%change: −1.387, 95% CI: −1.679,−1.094). For every quartile increase in the distance between the house and the water body, the risk of T2DM reduced by 11.5% (95% CI: 0.838,0.935), and the fasting blood glucose level was lowered by 1.371% (95% CI: −1.663%,−1.078%) in the fully adjusted model. The most protective effect was shown among people who lived more than five kilometers away from the nearest water body (OR: 0.843, 95% CI: 0.770,0.923). Associations between residential blue spaces and T2DM were not significant in the subgroup analysis (Table S2). Moreover, age, gender, BMI, physical activity, and monthly income did not modify the association between the blue space of the residence and the fasting blood glucose level (Table S3).

Sensitivity analysis confirmed the consistency and robustness of our results. After excluding some variants, such as participants taking hypoglycemic drugs or suffering from other chronic diseases, the results remained consistent (Table S4). Associations between the residential green space and the two health outcomes did not change substantially upon changing the 3-year exposure period to a 4-year, 2-year, or 1-year exposure period (Table S5). Additionally, we estimated the associations using different buffer radiuses, which were similar to the results using the 500 m buffer radius (Figure S2, Table S6).

## 4. Discussion

The results of the present study suggest that more residential green space may be beneficially associated with lower T2DM prevalence and fasting blood glucose levels. To our best knowledge, this is the first study to explore the association between residential blue space and T2DM. With the acceleration of urbanization and aging, our findings are of great significance for policymakers to facilitate the design of a suitable residential environment.

We found that the IQR increase in NDVI was associated with a 13.4% (95% CI: 9.7%,17.0%) decrease in the risk for T2DM. Our results are similar to results from a national health survey of 387,195 adults in the Netherlands, which discovered an 8% (95% CI: 7%,11%) decrease in the risk of T2DM, associated with an IQR increase of NDVI after adjustment for age, sex, BMI, and lifestyle characteristics [35]. A positive association between higher residential greenness and lower T2DM prevalence has also been reported in studies conducted in Australia [9], Canada [18], the United States [16], Germany [17], and China [13,14]. In addition, in a 20-year follow-up study in the UK, Dalton et al. showed that individuals living in the greenest neighborhood had a 19% lower relative hazard (hazard ratio (HR): 0.81, 95% CI: 0.67,0.99) of developing diabetes [36]. A Canadian cohort study involving 380,738 middle-aged and elderly people also reported similar results [19].

Negative associations between the abundance of residential green space and fasting blood glucose levels were identified, which is in agreement with results obtained in previous studies [20,37,38]. For the first time, we observed that the residential green space was associated with lower fasting blood glucose levels in men (%change, −2.060 in men vs. −0.972 in women) and the elderly (%change, −1.696 in elderly vs. −1.268 in young people). As relatively less research was conducted on blood glucose level associations, the results are mixed. Yang et al. and Danielle et al. reported results consistent with our findings that the men and the elderly benefited more from the protective effect of residential greenness than the general population [14,39]. However, few significant interactions were found between certain demographic characteristics and green space levels in two Asian studies [12,13].

We observed that the association between residential green space and T2DM was more significant in a smaller buffer zone in the sensitive analysis. Similar to our results, Cohen et al. reported that people living closer to the park tended to visit more often. More residents living within 0.5 miles of the park reported leisurely exercising five or more times per week more often than those living more than one mile away (49% vs. 35%) [40]. Yang et al. observed that the beneficial effects of greening were more obvious in the 500 m buffer zone, compared with the 1000 m buffer zone [41]. The optimal buffer radius setting is still inconclusive, but this may not affect the robustness of our results [42].

As an important landscape feature, the blue water body in the environment may be related to the health of residents, but there was still controversy about its impact. On the one hand, human beings have a hydrophilic nature, and water bodies can extend people’s stay time, thereby increasing the level of physical activity [43]. On the other hand, due to pollution, climate change and other reasons, the beauty of the waterscape is destroyed, and people’s perception of the beauty of the water body is reduced, which in turn has a negative impact on physical and mental health [44].

In our study, the prevalence of T2DM among residents who live farther from the blue space is lower than those who live closer. Since the research of residential blue space on T2DM prevalence is scarce, we have to compare our findings with other health outcomes. An American study found that living within 500 m of a freshwater body was associated with a higher birthweight of 10.1 g (95% CI: 2.0,18.2) [45]. Another investigation, conducted in Spain, showed that contact with green and blue spaces is beneficial to the behavioral development of school children [46]. However, several other studies reported no significant association between blue space and mental health [47,48,49]. We hope that more studies will focus on the association between blue space availability and T2DM prevalence in the future.

Three main mechanisms underlying the associations of the residential environment with T2DM and fasting blood glucose levels were identified earlier [50]. First, higher green space levels were associated with lower air pollution, since a large amount of green vegetation can effectively absorb ambient particles to filter and purify the air [51,52]. The harmful effects of long-term exposure to air pollution, such as an elevated risk for T2DM development, were less pronounced in high-green areas [53]. Second, the residential green and blue space availability was associated with higher levels of physical activity and lower obesity risk of residents [54,55]. Obesity is known as a critical cause for the development of T2DM, and physical activity is essential for the regulation of blood glucose levels and their maintenance within the normal range [56]. Third, a good living environment contributed to better sleep quality and was regarded as an effective upstream-level intervention to reduce the public health burden of mental disorders [57,58]. Briefly, sleep quality and mental health may affect the motor, nervous, and endocrine systems of our body, which in turn underlies the association between residential green and blue spaces and the aforementioned two health outcomes [59,60].

Some limitations of our research need to be acknowledged. First, since we used baseline survey data from an established cohort, the causal inference was limited. Second, there is no urban level classification system in China, and we could only classify the participants according to residential addresses, which may be biased. Third, we did not measure glycated hemoglobin A1c (HbA1c), which reflects the long-term average blood glucose level of subjects [61]. Thus, it was difficult to estimate the association of residential environment with long-term glucose metabolism. Fourth, NDVI and EVI were obtained based on the coded residential location from remote sensing; the top-down satellite image was different from the human eye level scene. In future research, we will try to use Google Street View (GSV) and deep learning to calculate the Green Landscape Index (GVI), which refers to greenness from the visual perspective of pedestrians [62,63]. Furthermore, since we did not have information on the traffic and noise, their potential confounding effects were not accounted for in the study [64]. Nevertheless, with the light traffic burden and lower population density in rural areas, traffic and noise might not have had considerable impacts on our results.

## 5. Conclusions

We observed that more abundant residential green space was associated with a lower risk of T2DM and decreased fasting blood glucose levels in a Chinese population. Future research is needed to further clarify this association and fully understand its potential mechanism.

## Figures and Tables

**Table 1:** Basic characteristics of study participants.

Characteristics ^a^	No Type 2 Diabetes Mellitus	Type 2 Diabetes Mellitus	Total
N	35,345 (90.6)	3674 (9.4)	39,019 (100.0)
NDVI (unit) *	0.48 ± 0.07	0.47 ± 0.07	0.48 ± 0.07
EVI (unit) *	0.34 ± 0.06	0.33 ± 0.06	0.34 ± 0.06
DNW (km) *	3.66 ± 2.71	3.41 ± 2.53	3.64 ± 2.70
FBG (mmol/L) *	5.19 ± 0.58	8.95 ± 2.86	5.54 ± 1.50
Age (years) *	55.10 ± 12.34	60.35 ± 9.29	55.58 ± 12.18
Age < 65	26,401 (74.7)	2366 (64.4)	28,767 (73.7)
Age ≥ 65	8944 (25.3)	1318 (35.6)	10,252 (26.3)
Sex			
Male	13,987 (39.6)	1394 (37.9)	15,381 (39.4)
Female	21,358 (60.4)	2280 (62.1)	23,638 (60.6)
BMI (kg/m^2^) *	24.69 ± 3.52	26.18 ± 3.67	24.83 ± 3.56
BMI < 25	19,619 (55.5)	1430 (38.9)	21,049 (53.9)
BMI ≥ 25	15,726 (44.5)	2244 (61.1)	17,970 (46.1)
Education level *			
Elementary school or below	15,424 (43.6)	2031 (55.3)	17,455 (44.7)
Middle school	14,346 (40.6)	1211 (33.0)	15,557 (39.9)
High school or above	5575 (15.8)	432 (11.8)	6007 (15.4)
Marital status			
Married/cohabiting	31,784 (89.9)	3255 (88.6)	35,039 (89.8)
Widowed/single/ divorced/separation	3561 (10.1)	419 (11.4)	3980 (10.2)
Monthly income *			
Low	12,464 (35.3)	1447 (39.4)	13,911 (35.7)
Medium	11,653 (33.0)	1181 (32.1)	12,834 (32.9)
High	11,228 (31.8)	1048 (28.5)	12,274 (31.5)
Smoking *			
Never	25,643 (72.5)	2766 (75.3)	28,409 (72.8)
Former	2794 (7.9)	372 (10.1)	3166 (8.1)
Current	6908 (19.5)	536 (14.6)	7444 (19.1)
Drinking *			
Never	27,265 (77.1)	2900 (78.9)	30,165 (77.3)
Former	1578 (4.5)	237 (6.5)	1815 (4.7)
Current	6502 (18.4)	537 (14.6)	7039 (18.0)
High-fat diet (≥75 g/day) *			
No	28,489 (80.6)	3076 (83.7)	31,565 (80.9)
Yes	6856 (19.4)	598 (16.3)	7454 (19.1)
Fruit and vegetable intake (≥ 500 g/day) *			
No	20,354 (57.6)	2358 (64.2)	22,712 (58.2)
Yes	14,991 (42.4)	1316 (35.8)	16,307 (41.8)
Physical activity *			
Low	11,142 (31.5)	1439 (39.2)	12,581 (32.2)
Medium	13,445 (38.0)	1300 (35.4)	14,745 (37.8)
High	10,758 (30.4)	935 (25.4)	11,693 (30.0)
Family history of diabetes *			
No	34,083 (96.4)	3309 (90.1)	37,392 (95.8)
Yes	1262 (3.6)	365 (9.9)	1627 (4.2)

Abbreviations: NDVI, Normalized Difference Vegetation Index; EVI, Enhanced Vegetation Index; DNW, distance to the nearest water body; FBG, fasting blood glucose. ^a^ Data are expressed as mean ± standard deviation for continuous variables and number (percentage) for categorical variables; *t*-test was conducted for continuous variables and chi-squared tests for categorical variables. * Statistically significant difference (*p* < 0.001).

**Table 2:** Results of regression models for associations between residential environment and type 2 diabetes mellitus and fasting blood glucose levels.

	Type 2 Diabetes Mellitus or (95%CI)			Fasting Blood Glucose Levels %Change (95%CI)		
	Crude	Model 1	Model 2	Crude	Model 1	Model 2
**Residential green space**						
NDVI						
Continuous (per IQR)	0.810 (0.780,0.842) *	0.846 (0.813,0.880) *	0.866 (0.830,0.903) *	−1.962 (−2.292,−1.631) *	−1.677 (−2.008,−1.345) *	−1.384 (−1.726,−1.040) *
Q1: <0.449	Reference	Reference	Reference	Reference	Reference	Reference
Q2: 0.449–0.499	0.919 (0.840,1.006)	0.923 (0.841,1.012)	0.913 (0.832,1.003)	2.125 (1.313,2.944)	2.259 (1.449,3.075)	2.170 (1.359,2.987)
Q3: 0.499–0.533	0.752 (0.684,0.825) *	0.776 (0.706,0.855) *	0.802 (0.727,0.884)	−2.166 (−2.942,−1.385) *	−1.913 (−2.687,−1.133) *	−1.561 (−2.349,−0.767) *
Q4: >0.533	0.571 (0.516,0.631) *	0.641 (0.578,0.710) *	0.675 (0.606,0.751) *	−3.671 (−4.439,−2.896) *	−3.000 (−3.773,−2.221) *	−2.470 (−3.269,−1.665) *
EVI						
Continuous (per IQR)	0.800 (0.764,0.837) *	0.834 (0.796,0.874) *	0.858 (0.817,0.901) *	−1.905 (−2.291,−1.517) *	−1.620 (−2.007,−1.232) *	−1.273 (−1.672,−0.871) *
Q1: <0.306	Reference	Reference	Reference	Reference	Reference	Reference
Q2: 0.306–0.346	0.902 (0.823,0.989)	0.899 (0.818,0.987)	0.892 (0.810,0.981)	1.499 (0.685,2.320)	1.629 (0.817,2.448)	1.573 (0.759,2.393)
Q3: 0.346–0.392	0.758 (0.691,0.831) *	0.779 (0.709,0.857) *	0.803 (0.729,0.884)	−1.993 (−2.754,−1.226) *	−1.693 (−2.454,−0.925) *	−1.402 (−2.173,−0.625) *
Q4: >0.392	0.622 (0.565,0.685) *	0.676 (0.613,0.746) *	0.713 (0.643,0.790) *	−3.803 (−4.549,−3.052) *	−3.336 (−4.084,−2.582) *	−2.770 (−3.545,−1.989) *
**Residential blue space**						
Distance to the nearest water body						
Continuous (per IQR)	0.884 (0.837,0.934) *	0.883 (0.836,0.933) *	0.885 (0.838,0.935) *	−1.387 (−1.679,−1.094) *	−1.384 (−1.676,−1.091) *	−1.371 (−1.663,−1.078) *
<2 km	Reference	Reference	Reference	Reference	Reference	Reference
2–5 km	0.999 (0.919,1.087)	0.998 (0.917,1.086)	0.993 (0.912,1.081)	−0.608 (−1.096,−0.118)	−0.613 (−1.102,−0.123)	−0.635 (−1.123,−0.145)
>5 km	0.842 (0.770,0.922) *	0.842 (0.769,0.921) *	0.843 (0.770,0.923) *	−1.840 (−2.347,−1.330) *	−1.839 (−2.346,−1.329) *	−1.829 (−2.335,−1.320) *

Crude model: no adjustment. Adjusted Model 1: adjusted for age, sex, body mass index, monthly income, and physical activity. Adjusted Model 2: also adjusted for education level, marital status, smoking, drinking, high-fat diet, fruit and vegetable intake, and family history of diabetes. * Statistically significant association (*p* < 0.001).

**Table 3:** Interaction effects of the association between residential green space and type 2 diabetes mellitus and fasting blood glucose levels.

Group	Type 2 Diabetes Mellitus				Fasting Blood Glucose Levels			
	NDVI		EVI		NDVI		EVI	
	OR (95%CI)	*P*_-interaction_	OR (95%CI)	*P*_-interaction_	%Change (95%CI)	*P*_-interaction_	%Change (95%CI)	*P*_-interaction_
Age (years)								
<65	0.880 (0.837,0.926)		0.875 (0.825,0.929)		−1.268 (−1.614,−0.921)		−1.109 (−1.513,−0.702)	
≥65	0.833 (0.777,0.892)	0.197	0.825 (0.760,0.895)	0.242	−1.696 (−2.056,−1.336)	<0.001	−1.696 (−2.124,−1.267)	<0.001
Sex								
Male	0.827 (0.774,0.884)		0.809 (0.747,0.875)		−2.060 (−2.600,−1.516)		−1.954 (−2.586,−1.318)	
Female	0.890 (0.845,0.937)	0.084	0.889 (0.836,0.945)	0.058	−0.972 (−1.402,−0.541)	<0.001	−0.859 (−1.360,−0.355)	0.004
BMI (kg/m^2^)								
<25	0.858 (0.821,0.895)		0.846 (0.804,0.891)		−1.410 (−1.760,−1.059)		−1.298 (−1.710,−0.884)	
≥25	0.872 (0.836,0.910)	0.083	0.868 (0.825,0.913)	0.071	−1.352 (−1.705,−0.997)	0.459	−1.242 (−1.659,−0.823)	0.621
Monthly income								
Low	0.861 (0.807,0.919)		0.857 (0.794,0.925)		−1.427 (−1.981,−0.869)			
Medium	0.857 (0.797,0.922)	0.927	0.843 (0.773,0.920)	0.782	−1.464 (−2.047,−0.879)	0.926	−1.372 (−2.058,−0.682)	0.783
High	0.881 (0.817,0.950)	0.646	0.876 (0.802,0.957)	0.713	−1.248 (−1.847,−0.646)	0.665	−1.206 (−1.906,−0.502)	0.942
Physical activity								
Low	0.860 (0.804,0.920)		0.846 (0.781,0.917)		−1.220 (−1.812,−0.624)			
Medium	0.837 (0.784,0.893)	0.562	0.827 (0.766,0.893)	0.678	−1.493 (−2.128,−0.854)	0.584	−1.304 (−1.916,−0.688)	0.561
High	0.925 (0.850,1.006)	0.182	0.930 (0.843,1.025)	0.141	−1.438 (−1.963,−0.911)	0.535	−1.496 (−2.231,−0.754)	0.369

## Data Availability

The data presented in this study are available on request from the corresponding author.

## Supplementary Materials

The following are available online at https://www.mdpi.com/2305-6304/9/1/11/s1, Table S1. Basic demographic and socioeconomic characteristics of study participants stratified by residential green space. Table S2. Interaction effects of the association between residential blue space and type 2 diabetes mellitus. Table S3. Interaction effects of the association between residential blue space and fasting blood glucose levels. Table S4. Sensitivity analyses for associations between residential green space and type 2 diabetes mellitus and fasting blood glucose levels. Table S5. Sensitivity analyses for associations between residential green space (different exposure time) and type 2 diabetes mellitus and fasting blood glucose levels. Table S6. Sensitivity analyses for associations between residential green space (different buffer radius) and type 2 diabetes mellitus and fasting blood glucose levels. Figure S1. The calculation process of blue space distance. Figure S2. Sensitivity analyses for associations between residential green space (different buffer radius) and type 2 diabetes mellitus and fasting blood glucose levels.
