# Supplementary material for: Residential Green and Blue Spaces and Type 2 Diabetes Mellitus: A Population-Based Health Study in China
Source: Toxics. 2021 Jan 16;9(1):11. doi: 10.3390/toxics9010011 (PMC7830986; doi:10.3390/toxics9010011)
Supplement: Supplementary file 1 [file toxics-09-00011-s001.pdf]

Supplementary Materials: Residential Green and Blue Spaces and Type 2 Diabetes Mellitus: A Population-Based Health Study in China

Ruijia Li, Gongbo Chen, Anqi Jiao, Yuanan Lu, Yuming Guo, Shanshan Li, Chongjian Wang and Hao Xiang

Table S1. Basic demographic and socioeconomic characteristics of study participants stratified by residential green space.

Characteristic [a]	Residential Green Space							
	Q1		Q2		Q3		Q4	
	Without T2DM	T2DM	Without T2DM	T2DM	Without T2DM	T2DM	Without T2DM	T2DM
N	8484 (89.0)	1051 (11.0)	8772 (89.0)	1080 (11.0)	8777 (90.8)	885 (9.2)	9312 (93.4)	658 (6.6)
NDVI (unit)	0.39 ± 0.04	0.39 ± 0.04	0.46 ± 0.01	0.46 ± 0.01	0.51 ± 0.01	0.51 ± 0.01	0.55 ± 0.01	0.55 ± 0.01
EVI (unit)	0.26 ± 0.04	0.26 ± 0.04	0.32 ± 0.01	0.32 ± 0.01	0.37 ± 0.02	0.37 ± 0.02	0.40 ± 0.02	0.40 ± 0.02
FBG (mmol/L)	5.22 ± 0.58	8.94 ± 2.91	5.30 ± 0.57	8.80 ± 2.67	5.20 ± 0.57	9.09 ± 2.96	5.05 ± 0.56	8.97 ± 2.87
DNW (km)	3.37 ± 2.56	3.12 ± 2.17	3.93 ± 3.00	3.84 ± 2.75	3.60 ± 2.88	3.18 ± 2.79	3.76 ± 2.32	3.58 ± 2.30
Age (years)	54.81 ± 12.31	60.39 ± 9.50	54.73 ± 12.46	60.70 ± 9.13	54.82 ± 12.92	60.68 ± 9.41	55.92 ± 11.64	59.118 ± 8.98
<65	6406 (75.5)	664 (63.2)	6612 (75.4)	680 (63.0)	6477 (73.8)	553 (62.5)	6906 (74.2)	469 (71.3)
≥65	2078 (24.5)	387 (36.8)	2160 (24.6)	400 (37.0)	2300 (26.2)	332 (37.5)	2406 (25.8)	189 (28.7)
Sex								
Male	3148 (37.1)	380 (36.2)	3331 (38.0)	408 (37.8)	3666 (41.8)	362 (40.9)	3842 (41.3)	244 (37.1)
Female	5336 (62.9)	671 (63.8)	5441 (62.0)	672 (62.2)	5111 (58.2)	523 (59.1)	5470 (58.7)	414 (62.9)
BMI (kg/m^2)	25.07 ± 3.54	26.45 ± 3.66	25.14 ± 3.56	26.42 ± 3.83	24.65 ± 3.54	26.08 ± 3.55	23.95 ± 3.31	25.48 ± 3.48
BMI < 25	4328 (51.0)	375 (35.7)	4413 (50.3)	390 (36.1)	4913 (56.0)	357 (40.3)	5965 (64.1)	308 (46.8)
BMI ≥ 25	4156 (49.0)	676 (64.3)	4359 (49.7)	690 (63.9)	3864 (44.0)	528 (59.7)	3347 (35.9)	350 (53.2)
Education level								
Elementary school or below	3555 (41.9)	576 (54.8)	3532 (40.3)	614 (56.9)	3807 (43.4)	498 (56.3)	4530 (48.6)	343 (52.1)
Middle school	3655 (43.1)	352 (33.5)	3506 (40.0)	339 (31.4)	3412 (38.9)	287 (32.4)	3773 (40.5)	233 (35.4)
High school or above	1274 (15.0)	123 (11.7)	1734 (19.8)	127 (11.8)	1558 (17.8)	100 (11.3)	1009 (10.8)	82 (12.5)
Marital status								
Married/cohabiting	7583 (89.4)	915 (87.1)	7941 (90.5)	958 (88.7)	7873 (89.7)	779 (88.0)	8387 (90.1)	603 (91.6)
Widowed/single/ divorced/separation	901 (10.6)	136 (12.9)	831 (9.5)	122 (11.3)	904 (10.3)	106 (12.0)	925 (9.9)	55 (8.4)
Monthly income								
Low	3121 (36.8)	440 (41.9)	2674 (30.5)	392 (36.3)	2946 (33.6)	344 (38.9)	3723 (40.0)	271 (41.2)
Medium	2930 (34.5)	331 (31.5)	3310 (37.7)	390 (36.1)	2705 (30.8)	281 (31.8)	2708 (29.1)	179 (27.2)
High	2433 (28.7)	280 (26.6)	2788 (31.8)	298 (27.6)	3126 (35.6)	260 (29.4)	2881 (30.9)	208 (31.6)
Smoking								
Never	6266 (73.9)	808 (76.9)	6502 (74.1)	810 (75.0)	6209 (70.7)	645 (72.9)	6666 (71.6)	503 (76.4)
Former	744 (8.8)	120 (11.4)	693 (7.9)	119 (11.0)	680 (7.7)	73 (8.2)	677 (7.3)	60 (9.1)
Current	1474 (17.4)	123 (11.7)	1577 (18.0)	151 (14.0)	1888 (21.5)	167 (18.9)	1969 (21.1)	95 (14.4)
Drinking								
Never	6527 (76.9)	832 (79.2)	6685 (76.2)	846 (78.3)	6720 (76.6)	705 (79.7)	7333 (78.7)	517 (78.6)
Former	296 (3.5)	77 (7.3)	317 (3.6)	65 (6.0)	430 (4.9)	49 (5.5)	535 (5.7)	46 (7.0)
Current	1661 (19.6)	142 (13.5)	1770 (20.2)	169 (15.6)	1627 (18.5)	131 (14.8)	1444 (15.5)	95 (14.4)
High-fat diet (≥75g/day)								
No	6707 (79.1)	856 (81.4)	7414 (84.5)	949 (87.9)	7138 (81.3)	769 (86.9)	7230 (77.6)	502 (76.3)
Yes	1777 (20.9)	195 (18.6)	1358 (15.5)	131 (12.1)	1639 (18.7)	116 (13.1)	2082 (22.4)	156 (23.7)
Fruit and vegetable intake (≥500g/day)								
No	6159 (72.6)	824 (78.4)	6470 (73.8)	816 (75.6)	4208 (47.9)	466 (52.7)	3517 (37.8)	252 (38.3)
Yes	2325 (27.4)	227 (21.6)	2302 (26.2)	264 (24.4)	4569 (52.1)	419 (47.3)	5795 (62.2)	406 (61.7)
Physical activity								
Low	2709 (31.9)	429 (40.8)	3332 (38.0)	487 (45.1)	2609 (29.7)	314 (35.5)	2492 (26.8)	209 (31.8)
Moderate	3245 (38.2)	384 (36.5)	2638 (30.1)	302 (28.0)	3444 (39.2)	334 (37.7)	4118 (44.2)	280 (42.6)

High	2530 (29.8)	238 (22.6)	2802 (31.9)	291 (26.9)	2724 (31.0)	237 (26.8)	2702 (29.0)	169 (25.7)
Family history of diabetes								
No	8081 (95.2)	945 (89.9)	8381 (95.5)	924 (85.6)	8520 (97.1)	816 (92.2)	9101 (97.7)	624 (94.8)
Yes	403 (4.8)	106 (10.1)	391 (4.5)	156 (14.4)	257 (2.9)	69 (7.8)	211 (2.3)	34 (5.2)
Region								
Xuchang	5085 (59.9)	655 (62.3)	2863 (32.6)	385 (35.6)	171 (1.9)	20 (2.3)	3 (0.0)	0 (0.0)
Zhumadian	546 (6.4)	26 (2.5)	354 (4.0)	19 (1.8)	5139 (58.6)	400 (45.2)	8906 (95.6)	585 (88.9)
Kaifeng	633 (7.5)	92 (8.8)	983 (11.2)	128 (11.9)	603 (6.9)	68 (7.7)	-	-
Xinxiang	1371 (16.2)	180 (17.1)	4569 (52.1)	546 (50.6)	2864 (32.6)	397 (44.9)	403 (4.3)	73 (11.1)
Sanmenxia	849 (10.0)	98 (9.3)	3 (0.0)	2 (0.2)	-	-	-	-

Abbreviations: OR, odds ratio; CI, confidence interval; T2DM, type 2 diabetes mellitus; NDVI, Normalized Difference Vegetation Index; EVI, Enhanced Vegetation Index; FBG, fasting blood glucose; DNW, distance to the nearest water body. [a] Data are the mean ± standard deviation for continuous variables and number (percentage) for categorical variables.

Table S2. Interaction effects of the association between residential blue space and type 2 diabetes mellitus.

Group	Distance to the Nearest Water Body [a]					
	<2 km		2–5 km		>5 km	
	**OR (95%CI)**	***P*-interaction**	**OR (95%CI)**	***P*-interaction**	**OR (95%CI)**	***P*-interaction**
Age (years)						
<65	1.192(1.020,1.393)		0.994(0.860,1.149)		0.845(0.741,0.964)	
≥65	1.280(1.024,1.600)	0.573	0.962(0.770,1.202)	0.803	0.970(0.812,1.158)	0.151
Sex						
Male	1.403(1.149,1.713)		0.881(0.729,1.065)		0.899(0.773,1.045)	
Female	1.123(0.955,1.320)	0.056	1.049(0.900,1.223)	0.138	0.850(0.740,0.977)	0.507
BMI (kg/m^2)						
BMI < 25	1.214(1.018,1.448)		0.967(0.823,1.137)		0.854(0.740,0.984)	
BMI ≥ 25	1.227(1.028,1.465)	0.923	0.998(0.835,1.192)	0.789	0.888(0.768,1.027)	0.632
Monthly income						
Low	1.193(0.984,1.447)		1.154(0.950,1.403)		0.846(0.720,0.996)	
Medium	1.306(1.060,1.608)	0.497	0.749(0.609,0.922)	0.002	0.885(0.754,1.040)	0.645
High	1.155(0.918,1.453)	0.818	1.091(0.877,1.356)	0.693	0.879(0.736,1.049)	0.717
Physical activity						
Low	1.317(1.075,1.613)		1.021(0.824,1.265)		0.826(0.697,0.978)	
Medium	1.119(0.914,1.369)	0.221	0.932(0.776,1.119)	0.509	0.819(0.684,0.980)	0.933
High	1.234(0.986,1.545)	0.650	1.021(0.815,1.280)	0.999	0.968(0.814,1.150)	0.153

Abbreviations: OR, odds ratio; CI, confidence interval. [a] Models were adjusted for age, sex, body mass index, education level, marital status, monthly income, smoking, drinking, high-fat diet, fruit and vegetable intake, physical activity, family history of diabetes.

Table S3. Interaction effects of the association between residential blue space and fasting blood glucose levels.

Group	Distance to the Nearest Water Body [a]					
	<2 km		2–5 km		>5 km	
	**%change (95%CI)**	***P*-interaction**	**%change (95%CI)**	***P*-interaction**	**%change (95%CI)**	***P*-interaction**
Age (years)						
<65	1.003(0.102,1.913)		−0.518(−1.324,0.293)		−1.640(−2.276,−1.000)	
≥65	2.196(0.833,3.578)	0.124	−0.706(−2.018,0.624)	0.809	−1.375(−2.399,−0.339)	0.642
Sex						
Male	1.779(0.611,2.961)		−1.494(−2.574,−0.402)		−1.374(−2.179,−0.562)	
Female	1.094(0.139,2.059)	0.329	0.010(−0.875,0.903)	0.032	−1.780(−2.489,−1.066)	0.416
BMI (kg/m^2)						
<25	0.980(−0.030,1.999)		−0.794(−1.706,0.127)		−1.587(−2.295,−0.873)	
≥25	1.805(0.731,2.890)	0.225	−0.318(−1.353,0.728)	0.492	−1.628(−2.430,−0.818)	0.934

Monthly income						
Low	1.227(0.071,2.396)		0.361(−0.796,1.533)		−1.790(−2.621,−0.953)	
Medium	1.734(0.476,3.008)	0.535	−1.539(−2.722,−0.342)	0.023	−1.224(−2.116,−0.323)	0.331
High	1.121(−0.179,2.437)	0.899	−0.630(−1.822,0.577)	0.239	−1.812(−2.781,−0.833)	0.972
Physical activity						
Low	2.691(1.474,3.923)		−0.452(−1.770,0.884)		−1.787(−2.650,−0.916)	
Medium	0.147(−1.052,1.361)	0.002	−0.781(−1.803,0.251)	0.699	−1.908(−2.783,−1.026)	0.835
High	1.210(−0.097,2.535)	0.085	−0.370(−1.649,0.927)	0.929	−1.046(−1.998,−0.084)	0.234

Abbreviations: CI, confidence interval. [a] Models were adjusted for age, sex, body mass index, education level, marital status, monthly income, smoking, drinking, high-fat diet, fruit and vegetable intake, physical activity, family history of diabetes.

Table S4. Sensitivity analyses for associations between residential green space and type 2 diabetes mellitus and fasting blood glucose levels [a].

Green space	Type 2 Diabetes Mellitus OR (95%CI)	Fasting Blood Glucose Levels % Change (95% CI)
	All participants (n = 39,019)	
NDVI	0.866 (0.830,0.903)	−1.384 (−1.726,−1.040)
EVI	0.858 (0.817,0.901)	−1.273 (−1.672,−0.871)
	Exclude taking hypoglycemic drugs (n = 37,120)	
NDVI	0.875 (0.826,0.927)	−1.135 (−1.335,−0.935)
EVI	0.871 (0.814,0.932)	−1.102 (−1.334,−0.869)
	Exclude with diabetes family history (n = 37,392)	
NDVI	0.861 (0.824,0.899)	−1.264 (−1.502,−1.026)
EVI	0.853 (0.811,0.898)	−1.221 (−1.498,−0.944)
	Exclude hypertension (n = 26,287)	
NDVI	0.841 (0.794,0.891)	−1.256 (−1.527,−0.985)
EVI	0.833 (0.778,0.891)	−1.230 (−1.544,−0.915)
	Exclude dyslipidemias (n = 24,368)	
NDVI	0.839 (0.789,0.893)	−1.133 (−1.393,−0.873)
EVI	0.831 (0.772,0.893)	−1.076 (−1.379,−0.772)

Abbreviations: OR, odds ratio; CI, confidence interval; NDVI, Normalized Difference Vegetation Index; EVI, Enhanced Vegetation Index. [a] Models were adjusted for age, sex, body mass index, education level, marital status, monthly income, smoking, drinking, high-fat diet, fruit and vegetable intake, physical activity, family history of diabetes. Statistically significant association ($p < 0.001$).

Table S5. Sensitivity analyses for associations between residential green space (different exposure time) and type 2 diabetes mellitus and fasting blood glucose levels [a].

Green space	Median	IQR	Type 2 Diabetes Mellitus OR (95%CI)	Fasting Blood Glucose Levels % change (95% CI)
NDVI				
1-year average	0.495	0.085	0.866 (0.830,0.903)	−1.169 (−1.520,−0.817)
2-year average	0.495	0.089	0.845 (0.803,0.890)	−1.248 (−1.665,−0.830)
4-year average	0.498	0.100	0.849 (0.809,0.891)	−1.635 (−2.031,−1.237)
EVI				
1-year average	0.340	0.084	0.862 (0.823,0.904)	−1.331 (−1.714,−0.947)
2-year average	0.348	0.087	0.864 (0.829,0.900)	−1.397 (−1.733,−1.059)
4-year average	0.344	0.099	0.840 (0.796,0.886)	−1.539 (−1.972,−1.105)

Abbreviations: IQR, interquartile range; OR, odds ratio; CI, confidence interval; NDVI, Normalized Difference Vegetation Index; EVI, Enhanced Vegetation Index. [a] Models were adjusted for age, sex, body mass index, education level, marital status, monthly income, smoking, drinking, high-fat diet, fruit and vegetable intake, physical activity, family history of diabetes. Statistically significant association ($p < 0.001$).

Table S6. Sensitivity analyses for associations between residential green space (different buffer radius) and type 2 diabetes mellitus and fasting blood glucose levels [a].

Green space	Median	IQR	Type 2 Diabetes Mellitus OR (95%CI)	Fasting Blood Glucose Levels %Change (95%CI)
NDVI				
300 m buffer	0.518	0.119	0.860 (0.828,0.892)	−1.307 (−1.685,−0.928)
500 m buffer	0.516	0.117	0.866 (0.830,0.903)	−1.384 (−1.726,−1.040)
1000 m buffer	0.521	0.089	0.839 (0.810,0.869)	−1.237 (−1.552,−0.921)
3000 m buffer	0.503	0.093	0.889 (0.863,0.915)	−0.949 (−1.215,−0.682)
EVI				
300 m buffer	0.360	0.110	0.843 (0.811,0.875)	−1.280 (−1.633,−0.926)
500 m buffer	0.359	0.112	0.858 (0.817,0.901)	−1.273 (−1.672,−0.871)
1000 m buffer	0.356	0.088	0.860 (0.827,0.893)	−0.897 (−1.246,−0.546)
3000 m buffer	0.349	0.093	0.904 (0.873,0.936)	−0.708 (−1.024,−0.392)

Abbreviations: IQR, interquartile range; OR, odds ratio; CI, confidence interval; NDVI, Normalized Difference Vegetation Index; EVI, Enhanced Vegetation Index. [a] Models were adjusted for age, sex, body mass index, education level, marital status, monthly income, smoking, drinking, high−fat diet, fruit and vegetable intake, physical activity, family history of diabetes. Statistically significant association ($p < 0.001$).

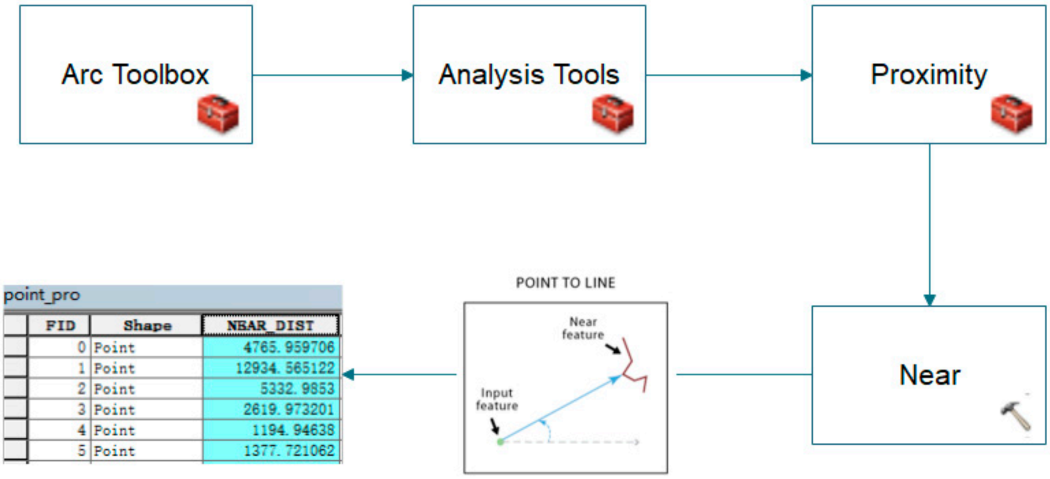

Figure S1. The calculation process of blue space distance.

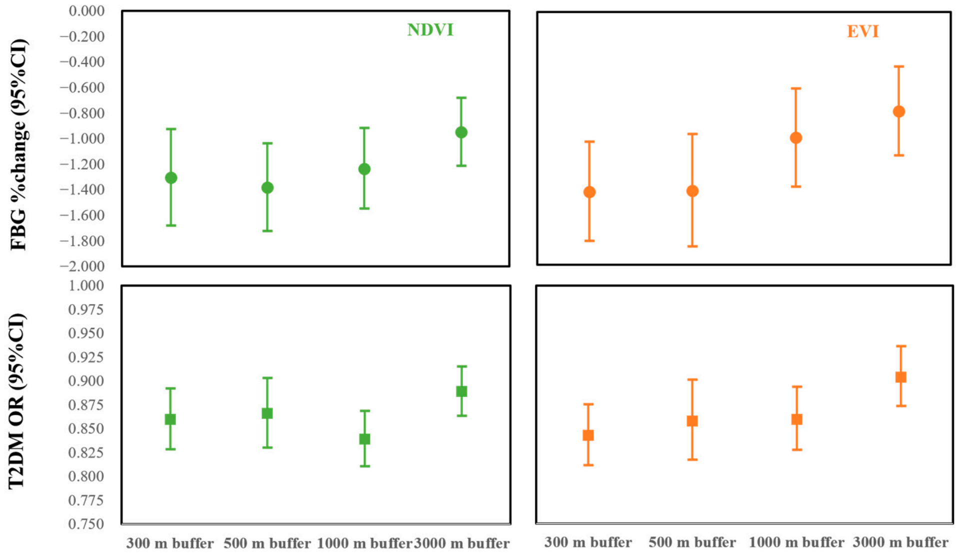

Figure S2. Sensitivity analyses for associations between residential green Scheme 2. diabetes mellitus and fasting blood glucose levels. Abbreviations: T2DM, type 2 diabetes mellitus; FBG, fasting blood glucose; OR, odds ratio; CI, confidence interval; NDVI, Normalized Difference Vegetation Index; EVI, Enhanced Vegetation Index. Models were adjusted for age, sex, body mass index, education level, marital status, monthly income, smoking, drinking, high−fat diet, fruit and vegetable intake, physical activity, family history of diabetes. Statistically significant association ($p < 0.001$).
